# Immunogenicity and safety of the booster COVID-19 vaccine among people with HIV: a systematic review and meta-analysis

**DOI:** 10.3389/fimmu.2025.1668576

**Published:** 2025-09-17

**Authors:** Zhenzhen Chen, Chunping Wan, Bing Chen, Qingyan Mo, Mingqian Ju, Kunlong Deng, Xiaohong Li, Dongdong Qin

**Affiliations:** ^1^ Airport Emergency Center, The Affiliated Guangdong Second Provincial General Hospital of Jinan University, Guangzhou, Guangdong, China; ^2^ The Fifth Affiliated Hospital, Yunnan University of Chinese Medicine, Kunming, Yunnan, China; ^3^ School of Medicine, Kunming University, Kunming, Yunnan, China; ^4^ Key Laboratory of Traditional Chinese Medicine for Prevention and Treatment of Neuropsychiatric Diseases, Yunnan University of Chinese Medicine, Kunming, Yunnan, China

**Keywords:** COVID-19, booster vaccines, HIV, immunogenicity, CD4+ T cell, seroconversion

## Abstract

**Background:**

Human immunodeficiency virus (HIV) and COVID-19 continue to pose significant global public health challenges. Although vaccination is essential for preventing COVID-19 in people with HIV (PWH), evidence on the immunogenicity and safety of booster doses remains limited. This systematic review aimed to assess the immunogenicity and safety of COVID-19 booster vaccination in PWH.

**Methods:**

We conducted a comprehensive literature search in PubMed, EMBASE, and the Cochrane Library. Eligible studies included PWH who had received three or more doses of a COVID-19 vaccine.

**Results:**

Across 54 included studies, 4,685 of 5,229 PWH achieved seroconversion following a third or subsequent COVID-19 vaccine dose—an improvement over rates observed after the primary vaccine series. In 23 studies comparing 2,284 PWH with 1,813 healthy controls (HC), no significant differences in seroconversion rates were found (p ≥ 0.05). Among PWH, 22 studies reported significantly higher seroconversion rates in individuals with CD4^+^ T cell counts >200 cells/mm³ compared to those with counts <200 cells/mm³. Booster vaccination enhanced CD4^+^ T cell responses to levels comparable to HC, although CD8^+^ T cell responses remained markedly lower. Five studies reported adverse events following booster doses, none of which were classified as serious.

**Conclusion:**

COVID-19 booster vaccination is effective in enhancing immune protection and reducing severe disease in PWH. Optimal vaccine dosing is especially important in individuals with low CD4^+^ T cell counts. Tailoring booster strategies may improve seroconversion and overall immune response in this population.

**Systematic review registration:**

https://www.crd.york.ac.uk/PROSPERO/, identifier CRD42024605151

## 1 Introduction

Although the World Health Organization has declared an end to the coronavirus disease 2019 (COVID-19) public health emergency, the prevalence of SARS-CoV-2 remains high (1). As of February 2, 2025, more than 770 million individuals worldwide have been infected, with over 7.08 million reported deaths (WHO COVID-19 Dashboard). The COVID-19 landscape is further complicated by acquired immunodeficiency syndrome (AIDS), a condition marked by profound immunodeficiency due to untreated human immunodeficiency virus (HIV) infection. People with HIV (PWH) experience a higher burden of non-HIV comorbidities, which predispose to more severe COVID-19, particularly in the setting of untreated HIV infection or low CD4^+^ T cell counts (2–4). Recent data also indicate that PWH have had a relative increase in mortality with COVID-19 compared with the general population (5).

Vaccination remains a cornerstone of COVID-19 control, and several vaccine platforms—mRNA (BNT162b2, mRNA-1273), adenoviral vector (ChAdOx1 nCoV-19, Ad26.COV2.S), and inactivated virus (BBIBP-CorV, CoronaVac)—were widely deployed during the pandemic. While numerous meta-analyses have examined primary vaccination outcomes in PWH, limited evidence exists regarding the immunogenicity and safety of COVID-19 booster doses in this population (6–9). Existing studies on primary vaccination show that PWH, across ethnic and geographic contexts, develop neutralizing antibodies and exhibit reduced COVID-19 risk post-vaccination (10). However, seroconversion rates remain lower in PWH compared to healthy controls (HC) (11). To maintain adequate protection, booster doses are recommended for immunocompromised individuals in many countries (12). Nonetheless, the magnitude and quality of humoral and T-cell responses to booster vaccination in PWH—particularly those with advanced immunosuppression—remain inadequately characterized.

Vaccine-induced protection depends not only on neutralizing antibodies but also on cellular immunity, particularly CD4+ T cell responses (13). CD4^+^ T cells, primary targets of HIV, are essential for orchestrating both humoral and cellular immunity. Regulatory T cells (Tregs) also contribute to immune modulation following vaccination (14). Diminished CD4^+^ T cell counts can impair neutralizing antibody production, lower seroconversion rates, and compromise overall vaccine efficacy.

This systematic review and meta-analysis evaluated the immunogenicity and safety of administering three or more COVID-19 vaccine doses to PWH. It compared seroconversion rates between PWH and HC, and assessed the association between neutralizing antibody levels and CD4^+^ T cell counts, along with other immune correlates. The findings offer critical insights for optimizing booster vaccination strategies in PWH.

## 2 Methods

This systematic review adhered to Preferred Reporting Items for Systematic Reviews and Meta-Analysis (PRISMA) guidelines (15) and was registered in PROSPERO (CRD42024605151).

### 2.1 Search strategy

We systematically searched PubMed, EMBASE, and the Cochrane Library for studies evaluating SARS-CoV-2 vaccine immunogenicity in PWH. The final search was conducted on October 22, 2024. Search terms included combinations of (“COVID-19” OR “Coronavirus” OR “SARS-CoV-2”), (“HIV” OR “Acquired Immunodeficiency Syndrome Virus” OR “PLWH”), and (“Vaccines” OR “Vaccination”). The complete search strategy is provided in **Supplementary Material**. Two reviewers (CZZ and LXH) independently screened titles, abstracts, and full texts; discrepancies were resolved by two additional reviewers (MQY and JMQ).

### 2.2 Inclusion and exclusion criteria

Studies were included if they met the following criteria: (1) observational studies (cohort, case-control, or cross-sectional), randomized controlled trials (RCTs), or non-randomized controlled trials; (2) involved PWH who had received ≥3 doses of a COVID-19 vaccine; and (3) reported extractable data on immunogenicity (humoral or cellular) or safety (local and systemic adverse reactions).

Exclusion criteria were as follows: (1) non-original studies (e.g., reviews, commentaries, or meta-analyses); (2) preprints and unpublished data; (3) unavailable full texts; and (4) studies involving only one or two vaccine doses.

### 2.3 Data extraction

Two authors (CZZ and LXH) independently extracted data using a standardized Excel form. Collected variables included: (1) basic study characteristics (first author, publication year, country, and study design); (2) participant demographics for PWH and HC (sample size, age, sex, and antiretroviral therapy [ART] status, viral load); (3) COVID-19 vaccine details (booster type and dosage, time since vaccination, and interval between primary and booster doses); (4) immunogenicity outcomes (e.g., number of PWH with neutralizing antibody seroconversion, anti-receptor binding domain IgG or anti-spike IgG levels, mean CD4^+^ T cell count, CD4^+^/CD8^+^ ratio, ART status, assay type, and cellular immunity assessment); and (5) safety outcomes based on reported adverse events in PWH.

### 2.4 Risk of bias assessment

Cohort and case-control studies were assessed using the Newcastle–Ottawa Quality Assessment Scale, while cross-sectional studies were evaluated using the Agency for Healthcare Research and Quality (AHRQ) criteria. Quality assessments were independently conducted by CZZ and LXH, with discrepancies resolved by WCP and CB.

### 2.5 Statistical analyses

Meta-analyses were performed using Review Manager Version 5.3 and STATA Version 15.1. For primary outcomes, relative risk (RR) and 95% confidence intervals (CI) were calculated using a random-effects model. RR values of <1 indicated lower seroconversion rates in vaccinated PWH compared to HC. Study heterogeneity was assessed using the I² statistic, with values ≥50% indicating substantial heterogeneity. Meta-regression and subgroup analyses were conducted to explore potential sources of heterogeneity. Sensitivity analyses were performed to evaluate the robustness of primary outcomes. Publication bias was assessed using funnel plots and Egger’s test.

## 3 Results

**Figure 1:**
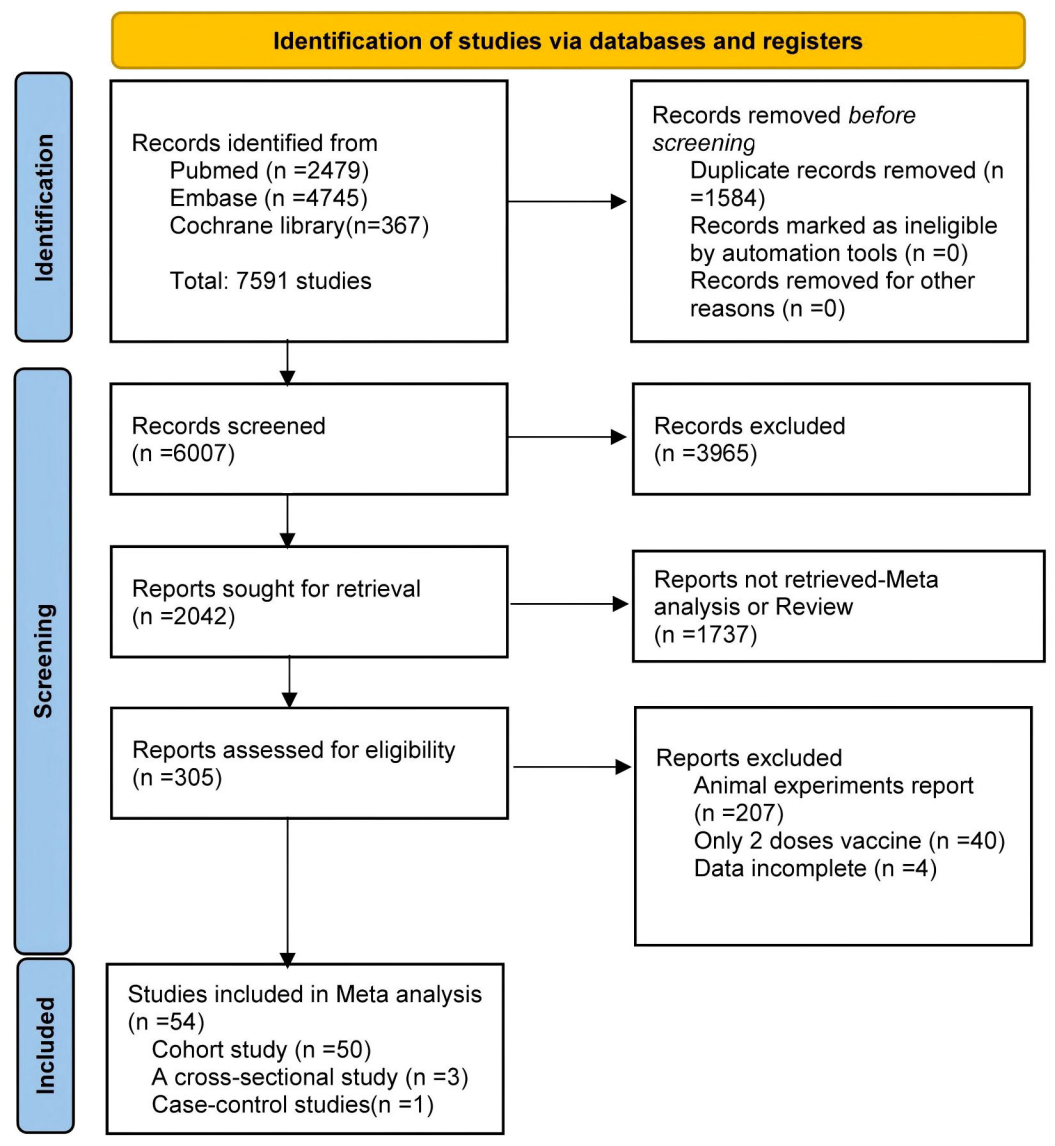
Flowchart of the article selection process.

The study selection process is summarized in **Figure 1**. Of the 7,591 articles retrieved, 1,584 duplicates were removed. After screening titles and abstracts, 3,965 irrelevant studies and 1,737 reviews or meta-analyses were excluded. Full-text review excluded 207 animal studies, 40 studies involving only two vaccine doses, and four studies with unusable data. Ultimately, 54 articles were included in the meta-analysis (16–48) 23 of which conducted direct comparisons between PWH and HC.

### 3.1 Characteristics of the included studies

Among the 54 studies included in this analysis, 33 (62.96%) evaluated mRNA vaccines (BNT162b2 or mRNA-1273), 15 (27.78%) assessed inactivated vaccines (BBIBP-CorV or CoronaVac), and one (1.85%) investigated an adenovirus vector vaccine (ChAdOx1). All of the PWH received ≥3 doses of a COVID-19 vaccine. Most studies were cohort designs (n = 50, 92.59%), followed by cross-sectional (n = 3, 5.56%) and case-control studies (n = 1, 1.85%). Geographically, 24 studies (44.44%) were conducted in Asia, 21 (38.89%) in Europe, and nine (16.67%) in North America. **Supplementary Table S2** provides detailed study characteristics.

Risk of bias assessment showed that 45 studies (83.33%) had a moderate risk, while five (9.26%) and four (7.41%) had low and high risks of bias, respectively (**Supplementary Tables S3**-**S5**).

### 3.2 Immune response rates in PWH

**Figure 2:**
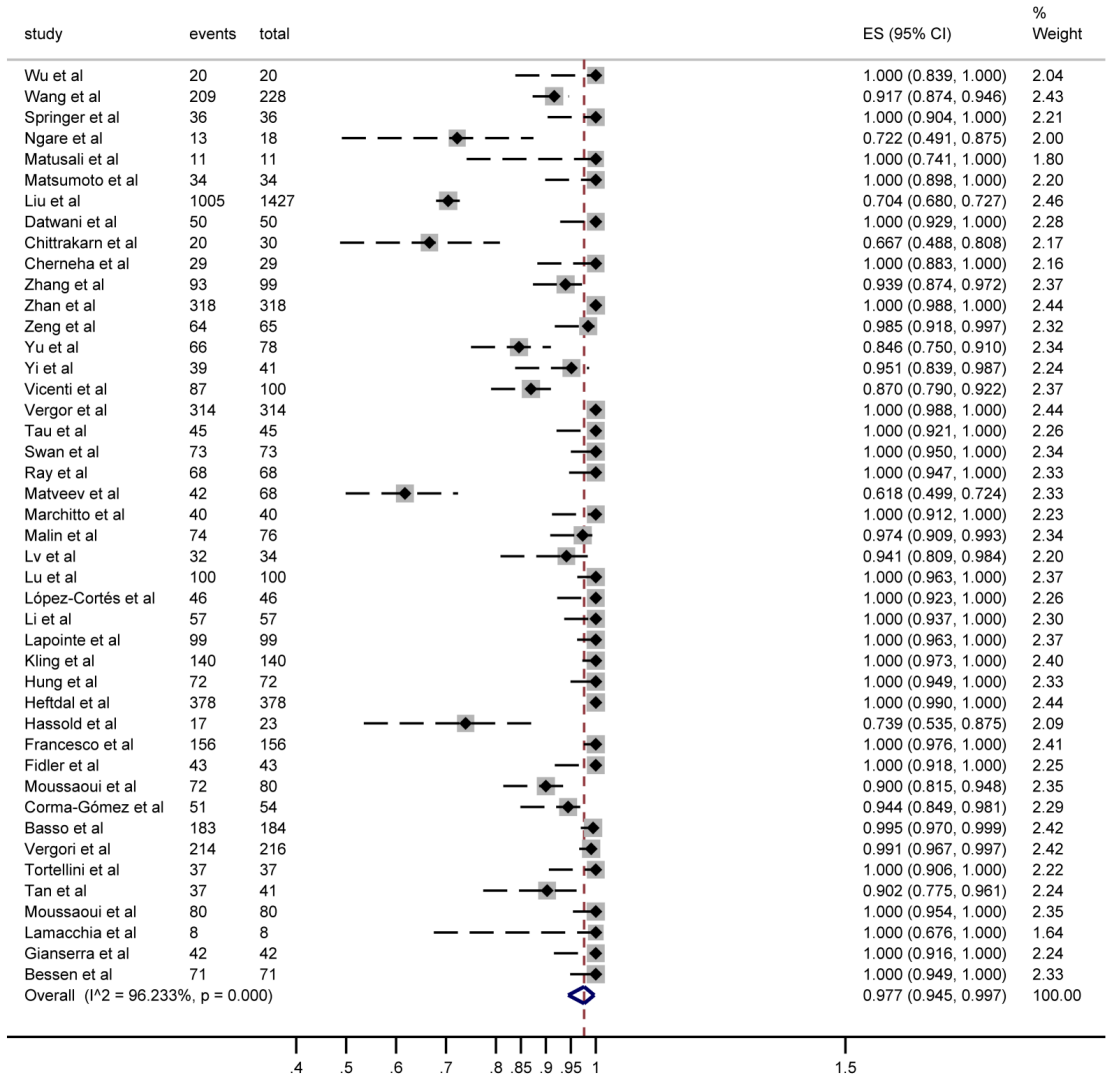
Forest plots of the included studies show the number of PWH seroconversions in each study (n, total number of PWHs; case, number of seroconversions in PWH).

Seroconversion outcomes were reported in 44 studies involving 5,229 PWH. After a third vaccine dose, 4,685 PWH seroconverted, yielding a pooled risk ratio (RR) of 0.977 (95% CI: 0.945–0.997) with substantial heterogeneity (I² = 96%; **Figure 2**).

### 3.3 Immune response comparison in PWH vs HC

**Figure 3:**
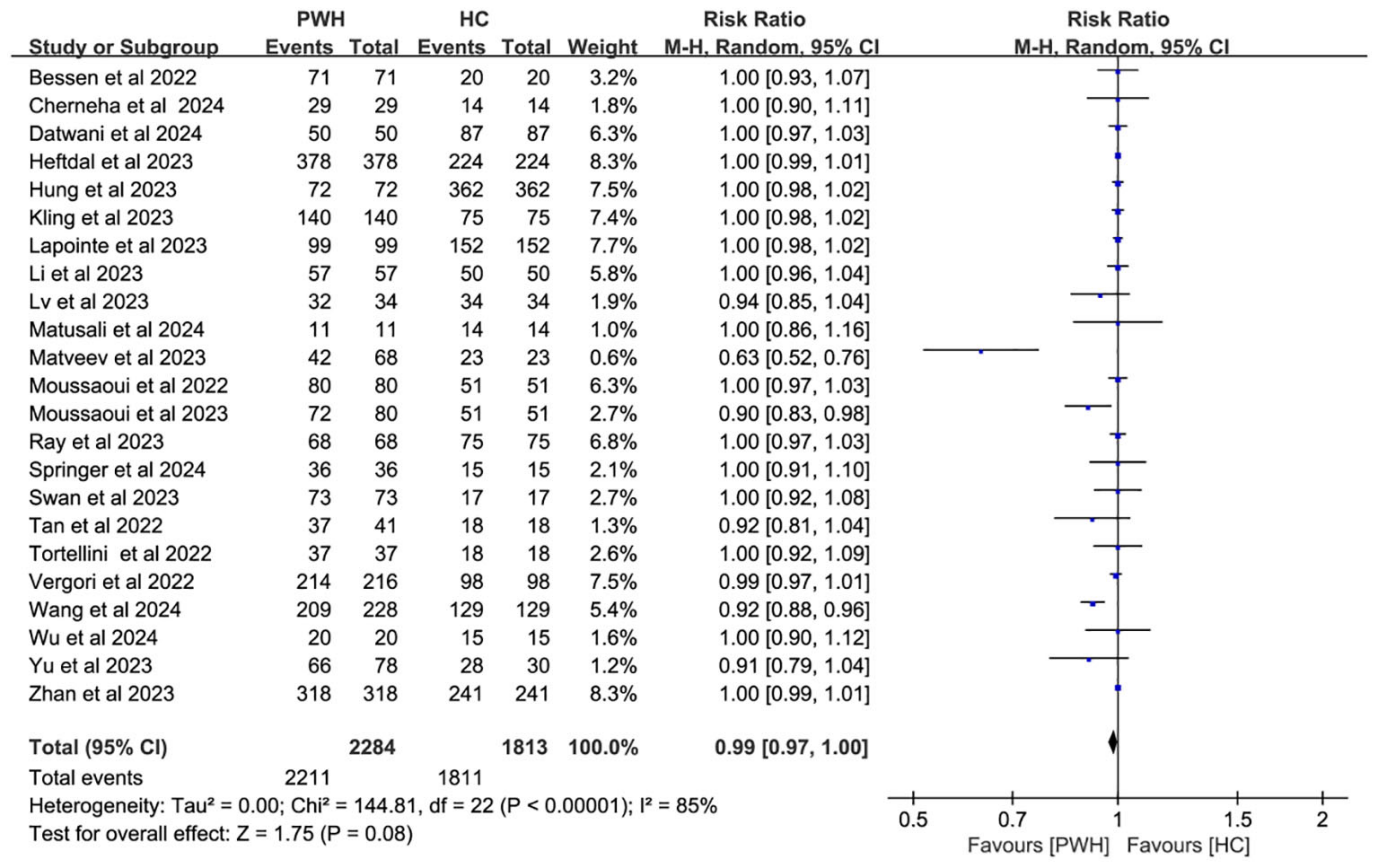
Risk ratios for seroconversion in PWH vs HC after the COVID-19 booster vaccine doses (CI, confidence interval; HC, healthy controls; M-H, Mantel–Haenszel; PWH, people with HIV).

In 23 studies comparing 2,284 PWH and 1,813 healthy controls (HC), seroconversion rates post–third dose were comparable between groups (RR = 0.99, 95% CI: 0.97–1.00), though heterogeneity remained considerable (I² = 85%; **Figure 3**, **Supplementary Table S3**).

**Figure 4:**
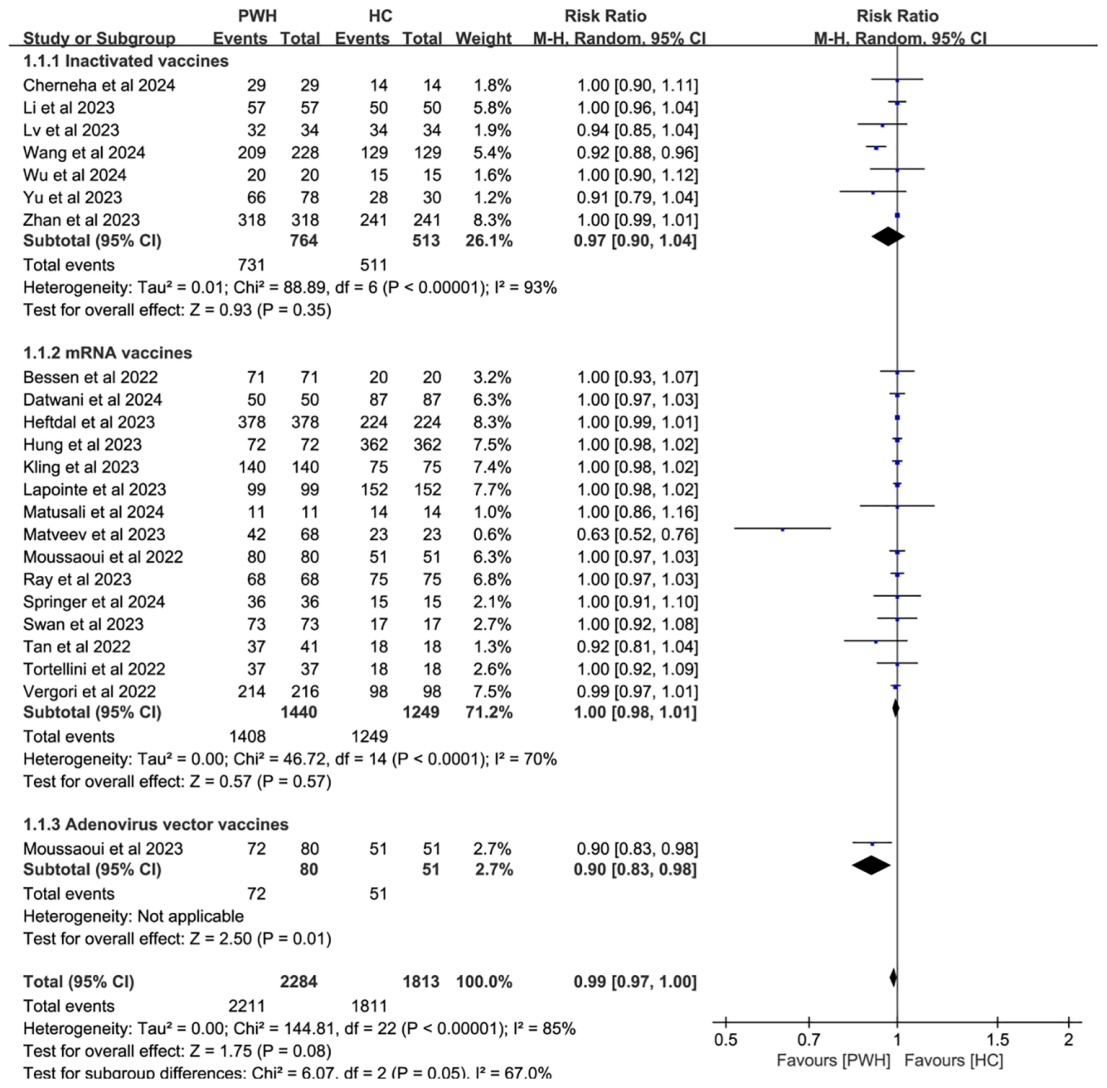
Subgroup analysis by vaccine type in PWH vs HC after booster COVID-19 vaccine doses (M-H, Mantel–Haenszel; PWH, people with HIV; HC, healthy controls; CI, confidence interval.).

**Figure 5:**
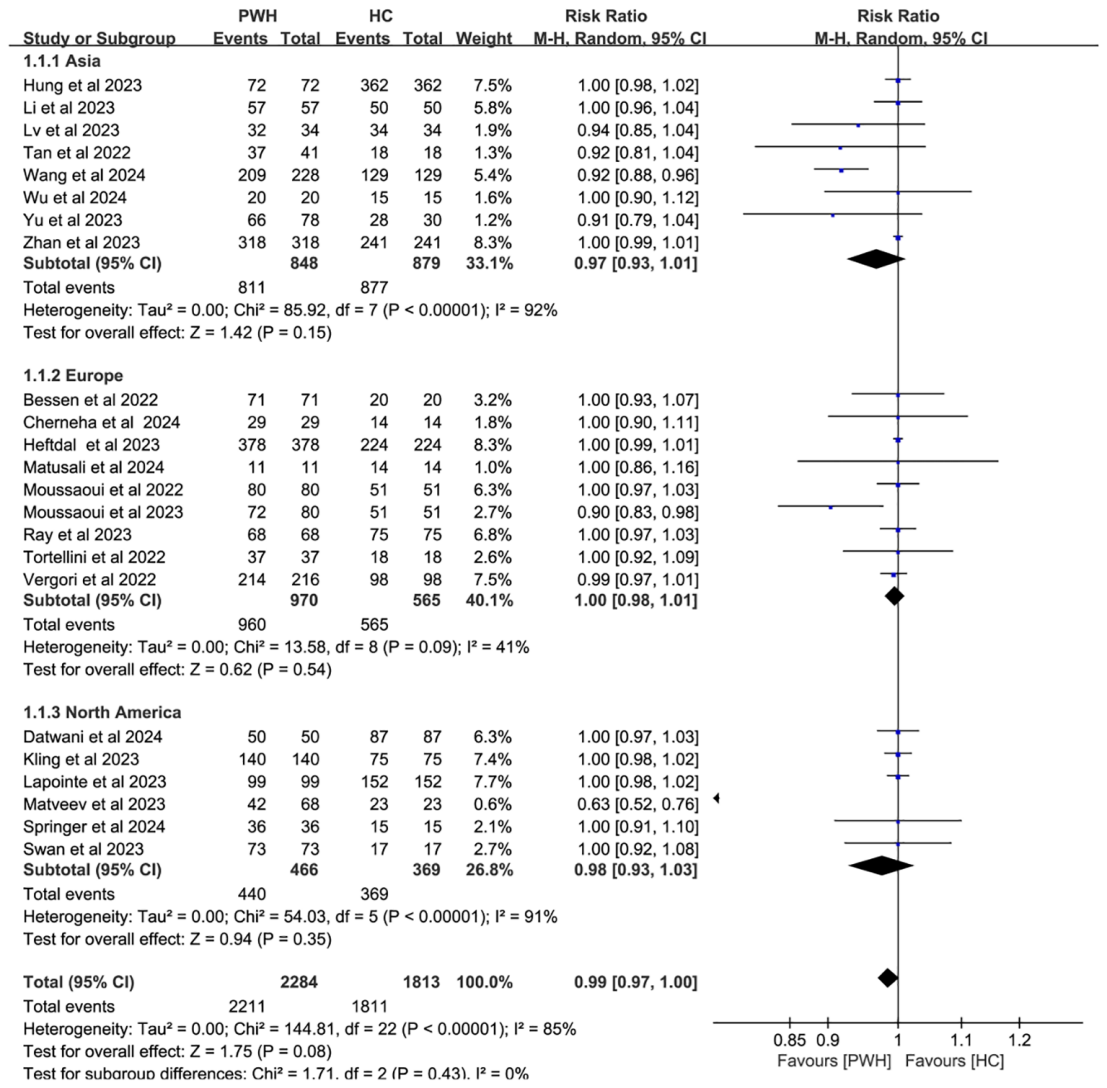
Subgroup analysis by continent in PWH vs HC after booster COVID-19 vaccine doses (CI, confidence interval; HC, healthy controls; M-H, Mantel–Haenszel; PWH, people with HIV).

Subgroup analyses by vaccine type and geographic region found no significant differences in seroconversion between PWH and HC (*p* ≥ 0.05). Notably, mRNA vaccines elicited immune responses in PWH that most closely resembled those in HC (RR = 1.00, 95% CI: 0.98–1.01; **Figure 4**). While regional variability may explain some heterogeneity (I² = 85%; **Figure 5**), evidence remains insufficient for definitive conclusions.

### 3.4 CD4^+^ cell immune responses in PWH

**Figure 6:**
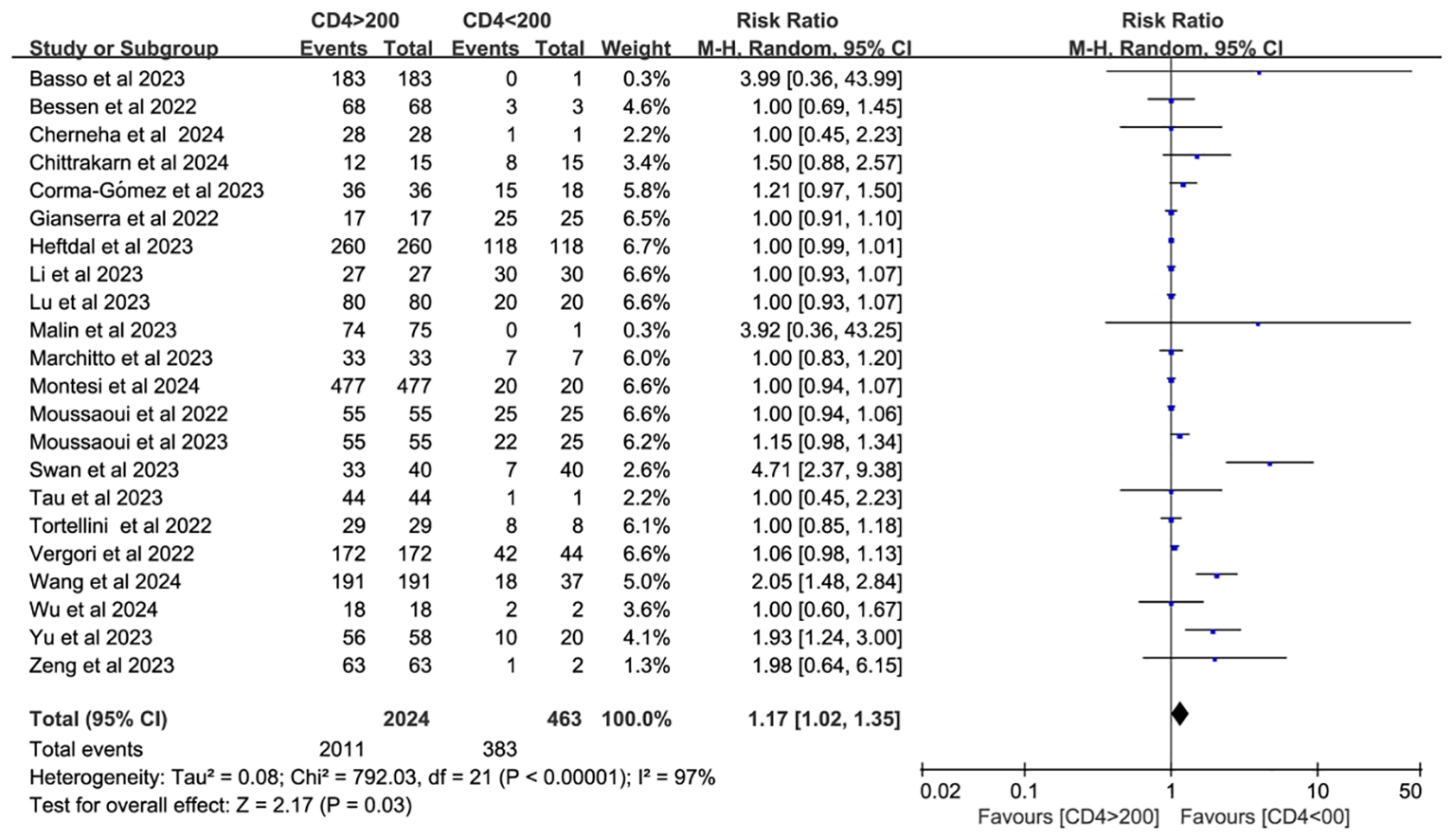
Risk ratio of PWH seroconversion after a booster dose of COVID-19 vaccine with 200 cells/mm³ CD4^+^ T cell count as the cutoff (CI, confidence interval; M-H, Mantel–Haenszel.).

**Figure 7:**
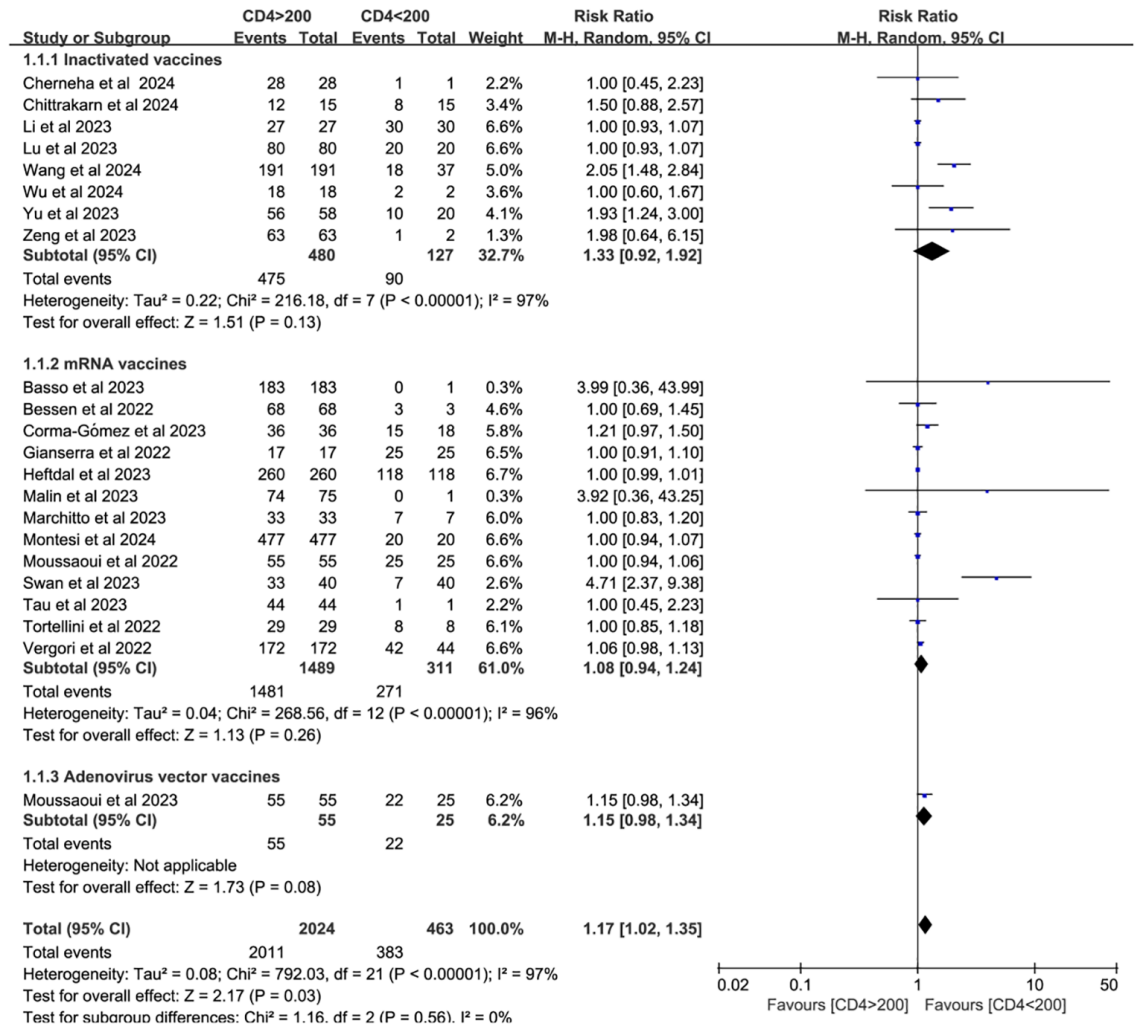
Subgroup analysis by vaccine type in PWH with CD4^+^ T cell counts of >200 cells/mm³ vs with <200 cells/mm³ after a booster COVID-19 vaccine dose (CI, confidence interval; M-H, Mantel–Haenszel).

**Figure 8:**
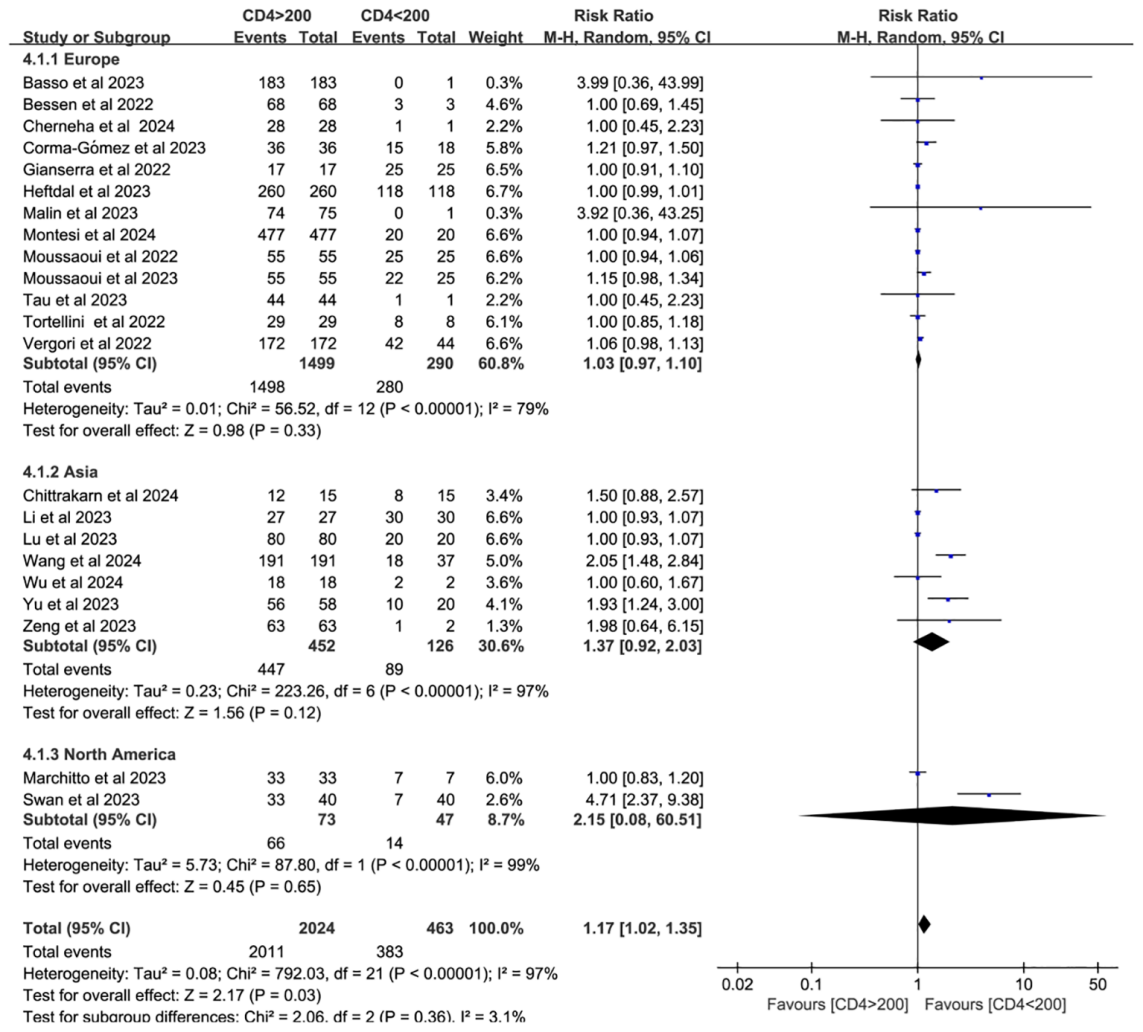
Subgroup analysis (based on continent) of PWH with CD4^+^ T-cell counts of >200 cells/mm³ vs <200 cells/mm³ after a booster COVID-19 vaccine dose (CI, confidence interval; M-H, Mantel–Haenszel).

Twenty-two studies analyzed CD4^+^ T cell–stratified seroconversion using a 200 cells/mm³ threshold. Among 2,487 PWH, 2,024 had CD4^+^ counts >200 cells/mm³ and 463 had <200 cells/mm³. Seroconversion was significantly lower in the <200 cells/mm³ group (*p* < 0.05; RR = 1.17, 95% CI: 1.02–1.35), with high heterogeneity (I² = 97%; **Figure 6**). Subgroup analyses confirmed that higher CD4^+^ counts favored better seroconversion outcomes (*p* < 0.05; **Figures 7**, **8**).

### 3.5 T-cell immune responses in PWH

T-cell immune responses were evaluated in 12 studies (17, 26, 29, 41, 44, 47, 49–54). Markers used to characterize CD4^+^ T cells included CD4^+^CD25^High^CD127^Low^ Tregs, OX40^+^/CD137^+^, CCR4, CD137, CD154, and cytokines such as TNF, IFN-γ, and IL-2. Activation of Treg and T follicular helper (Tfh) cells was also assessed. While CD8^+^ T cell responses in PWH receiving three doses were less robust than in HC, they were more durable than responses observed in PWH who received only one or two doses.

### 3.6 COVID-19 booster vaccine safety in PWH

Booster vaccine safety in PWH was assessed in five studies (18, 29, 33, 44, 55). Most adverse events occurred within seven days and resolved spontaneously. Local reactions—pain, swelling, redness, and itching—were reported in 17.10% of cases (13/76), while systemic events—drowsiness, myalgia, arthralgia, and nausea—occurred in 11.84% (9/76). No severe adverse events were reported. Two studies compared adverse reaction incidence between PWH (18.84%, 65/345) and non-HIV individuals (14.11%, 23/163) (18, 44). Although further statistical analysis was not feasible, the evidence supports the safety of booster vaccination in PWH.

### 3.7 Study quality

Quality assessments (**Supplementary Tables S3**-**S5**) indicated that 45 studies were of moderate quality, five of low quality, and four of high quality. Low quality was primarily attributed to small sample sizes and insufficient data on predefined endpoints.

### 3.8 Sensitivity analysis

Sensitivity analysis identified significant heterogeneity in Matveev’s study (**Supplementary Figure S1**). Systematic exclusion confirmed this study as a major contributor to overall heterogeneity (**Supplementary Figure S2**).

### 3.9 Publication bias

Funnel plot analysis and Egger’s test indicated no evidence of publication bias in immune response outcomes comparing PWH and HC following booster vaccination (*t* = –0.24, df = 23, *p* = 0.287). These findings suggest that PWH mount immune responses comparable to those of HC (**Supplementary Figure S3**).

## 4 Discussion

Given the proven efficacy of vaccination in reducing infection risk and disease severity among PWH, enhancing vaccine coverage in this population is imperative (9). This meta-analysis included 54 studies, with 44 studies assessing seroconversion in 5,229 PWH. Additionally, 23 studies comparing PWH to HC after a third vaccine dose found no significant difference in seroconversion rates between the two groups. Subgroup analyses by vaccine type and geographic region revealed that exclusion of the highly biased Matveev study substantially reduced heterogeneity. Consistent with previous findings, mRNA vaccines produced the highest seroconversion rates for booster doses (11, 56). These results suggest that a third vaccine dose elicits a humoral immune response in PWH comparable to that in HC, enhancing antibody production, which has been demonstrated by others to correlate with reduced disease severity (57) and hospitalization (58).

A significant correlation exists between neutralizing antibody levels and both the quantity and proportion of CD4^+^ T cells (59, 60). CD4^+^ T cells (helper T lymphocytes) are central to anti-HIV immunity but are also the primary targets of HIV infection. Therefore, monitoring CD4^+^ T cells can help assess the extent of immune damage and determine the effectiveness of antiretroviral therapy (ART) (61). As such, CD4^+^ T cell quantification is essential for staging HIV infection and AIDS, as well as monitoring treatment response (62). In SARS-CoV-2 infection, CD4^+^ T cell responses are more prominent than those of CD8^+^ T cells. Notably, SARS-CoV-2–specific CD4^+^ T cells demonstrate the strongest association with reduced COVID-19 severity compared to antibody or CD8^+^ T cell responses (14). A meta-analysis of individuals receiving one to two doses of hepatitis B virus (HBV) vaccine found that PWH with CD4^+^ T cell counts below 500 cells/mm^3^ exhibited diminished seroconversion responses to hepatitis B virus, although response rates were generally consistent across studies (63). To assess seroconversion after a third vaccine dose, this study stratified PWH by CD4^+^ T cell count using the 200 cells/mm³ threshold. The antibody conversion rate was significantly higher in those with counts above 200 cells/mm³ compared to those below this threshold. Although subgroup analyses by vaccine type and geographic region did not explain the heterogeneity, further analysis identified individuals with CD4^+^ T cell counts <200 cells/mm³ as the main source of heterogeneity. This heterogeneity may also be influenced by HIV disease progression and ART status.

T helper 1 (Th1) and follicular helper T (Tfh) cells—differentiated subsets of CD4^+^ T cells—are critical for establishing long-term antiviral immunity (64). Upon differentiation, these cells activate phagocytes and cytotoxic CD8^+^ T cells (65), and support B cell maturation in germinal centers to produce high-affinity, long-lived antibodies (66). Accordingly, CD4^+^ and Tfh cells are considered key markers of durable, protective antibody responses (67). CD4^+^ T cells produce a broad array of cytokines, including TNF-α, IFN-γ, IL-2, IL-4, IL-5, IL-9, IL-10, IL-17, and IL-22. Th1 cells predominantly secrete TNF-α, IFN-γ, and IL-2, while Th2 cells produce IL-4, IL-5, IL-9, and IL-13. Th17 and regulatory T cells (Tregs) are sources of IL-17 and IL-10, respectively. Notably, IL-22 is highly expressed by mucosal CD4^+^ T cells specific to SARS-CoV-2 (14, 17, 29, 37, 41, 44, 51). Findings from included studies showed that in PWH, a third vaccine dose activated Th1, Treg, and Tfh cells and increased cytokine expression—including IFN-γ, IL-17, and IL-22 (17, 41). Both CD4^+^ and CD8^+^ T cells contributed to enhanced antibody responses, with CD4^+^ T cells showing a more pronounced role in SARS-CoV-2 immunity (29, 44, 49, 66, 68, 69).

This study also found no differences in the safety profile of COVID-19 booster vaccines between PWH and HC. Previous research suggests that PWH experience fewer adverse events after the second dose compared to the first (70–72). However, limited data exist on the safety of booster doses, and individuals who tolerate initial doses well may be more likely to receive boosters (73, 74). Our findings support the safety of booster vaccination in PWH and may help reduce vaccine hesitancy in this population.

To the best of our knowledge, this is the most comprehensive analysis of COVID-19 booster vaccine immunogenicity and safety in PWH. The study included all relevant literature published from January 1, 2020, to October 22, 2024, involving PWH who received ≥3 doses of a COVID-19 vaccine. These findings provide valuable evidence to address booster vaccine hesitancy among PWH.

Nonetheless, this study has limitations. Firstly, we did not evaluate the real-world effectiveness of booster doses in PWH due to a lack of original studies. Taking into account various factors, such as ART, previous COVID-19 infection, the timing of vaccination, and other viral infections, further research should be designed to explicitly evaluate the effectiveness of booster vaccination in this population. Secondly, although seroconversion indicates potential protection against SARS-CoV-2, it is not a definitive clinical endpoint, which would be better assessed by infection rates. However, few studies address this outcome. Lastly, substantial heterogeneity across studies—likely due to differences in study location, timing, and sample size—underscores the need for future research to validate these findings.

## 5 Conclusion

The findings of our research suggest that the humoral immune response elicited in PWH following administration of the COVID-19 booster vaccine is comparable to that observed in HC. The enhanced antibody response following the administration of a booster shot may similarly contribute to this protective effect. However, PWH with CD4^+^ T cell counts below 200 cells/mm³ continue to exhibit suboptimal antibody seroconversion despite receiving booster doses. These results underscore the importance of booster vaccination in PWH, particularly those with advanced immunosuppression, and suggest that optimizing booster regimens may improve immune outcomes in this population.

## Data Availability

The datasets presented in this study can be found in online repositories. The names of the repository/repositories and accession number(s) can be found in the article/**Supplementary Material**.
