# Supplementary material for: Immunogenicity and safety of the booster COVID-19 vaccine among people with HIV: a systematic review and meta-analysis
Source: Front Immunol. 2025 Sep 17;16:1668576. doi: 10.3389/fimmu.2025.1668576 (PMC12484232; doi:10.3389/fimmu.2025.1668576)
Supplement: Supplementary file 1 [file Table1.docx]

**Supplementary Materials**

**Supplementary Table S1. The detailed search strategy.**

**Coverage: from the inception to October 22, 2024**

| **Database** | **Result** | **Search strings** |
| --- | --- | --- |
| Pubmed | 2479 | (COVID 19[mesh Terms]) OR (2019 ncov[mesh Terms]) OR (SARS-cov-2[mesh Terms]) OR (2019 ncov Disease)) OR (Coronavirus)) OR (Coronavirus Infections)) OR (COVID 19 Virus Disease)) OR (Disease, COVID-19 Virus)) OR (SARS Coronavirus 2 Infection) OR (COVID 19 Virus Infection)) OR (COVID 19 Pandemic)  AND  (HIV [mesh Terms]) OR (HIV Infections [mesh Terms]) OR (Human Immunodeficiency Virus) OR (Immunodeficiency Virus, Human) OR (Viruses, Human Immunodeficiency) OR (AIDS Virus) OR (Acquired Immunodeficiency Syndrome Virus) OR (PLWH)) OR (HIV-1)) OR (HIV-2)  AND  (vaccines [mesh Terms]) OR (vaccination [mesh Terms]) OR (Vaccine) |
| EMBASE | 4745 | #1. 'Covid 19'/exp OR 'sars-cov-2'/exp OR 'coronavirus infection'/exp OR 'coronavirinae'/exp OR 'coronavirus disease 2019'/exp OR 'severe acute respiratory syndrome coronavirus 2'/exp OR 'coronavirinae' OR 'coronavirus infection' OR 'coronavirus disease 2019' OR 'severe acute respiratory syndrome coronavirus 2' OR 'sars-cov*' OR 'coronavir*' OR 'covid*'  #2. 'Hiv infections'/exp OR 'hiv'/exp OR 'human immunodeficiency virus infection'/exp OR 'immunodeficiency virus'/exp OR 'hiv':ti, ab OR 'human immunodeficiency virus':ti, ab OR 'human immuno-deficiency virus':ti, ab OR 'human immune-deficiency virus':ti, ab OR 'acquired immune-deficiency syndrome':ti, ab OR 'acquired immunodeficiency syndrome':ti, ab OR 'plwh':ti, ab  #3. 'Vaccination'/exp OR 'vaccine'/exp OR 'vaccine*'  #4. #1 AND #2 AND #3 |
| Cochrane Library | 367 | #1 mesh descriptor: [COVID-19] explode all trees  #2 mesh descriptor: [SARS-cov-2] explode all trees  #3 #1 or #2  #4 (2019 ncov or 2019 ncov Disease or Coronavirus or Coronavirus Infections or COVID 19 Virus Disease or Disease, COVID-19 Virus or SARS Coronavirus 2 Infection or COVID 19 Virus Infection or COVID 19 Pandemic):ti, ab, kw (Word variations have been searched)  #5 #3 or #4  #6 mesh descriptor: [HIV] explode all trees  #7 mesh descriptor: [HIV Infections] explode all trees  #8 #6 or #7  #9 (Human Immunodeficiency Virus or Immunodeficiency Virus, Human or Viruses, Human Immunodeficiency or AIDS Virus or Acquired Immunodeficiency Syndrome Virus or PLWH or HIV-1 or HIV-2): ti, ab, kw (Word variations have been searched)  #10 #8 or #9  #11 mesh descriptor: [Vaccines] explode all trees  #12 mesh descriptor: [Vaccination] explode all trees  #13 #11 or #12  #14 (Vaccine): ti, ab, kw (Word variations have been searched)  #15 #13 or #14  #16 #5 and #10 and #15 |

**Supplementary Table S2. Characteristics of included studies.**

| **Author** | **Year** | **Country** | **Study design** | **PWH/n** | **HC/n** | **Age** | | **Gender F/M** | | **Booster vaccine type** | **Vaccine dose** | **Days after vaccination (median (IQR))** | **Interval days between booster and primary vaccination (median (IQR))** | **CD4 count (cells/μL, median (IQR))** | **CD4+/CD8+ T-cell ratio (median (IQR))** | **ART** | **Viral load (copies/mL, median (****IQR))** |
| --- | --- | --- | --- | --- | --- | --- | --- | --- | --- | --- | --- | --- | --- | --- | --- | --- | --- |
|  |  |  |  |  |  | PWH | HC | PWH | HC |  |  |  |  |  |  |  |  |
|  |  |  |  |  |  | median (IQR) | median (IQR) |  |  |  |  |  |  |  |  |  |  |
| Wu *et al.* | 2024 | China | Cohort study | 20 | 15 | 42 (37, 50) | 31 (29, 34) | 7/13 | 11/4 | Coronavac | 3 | 90 (NP) | 210 (NP) | 431.5 (81.25, 562.25) | NP | 75% | NP |
| Wang *et al.* | 2024 | China | Cohort study | 45 | 29 | 35 (31, 38) | 41 (35.50, 48.50) | 2/43 | 6/23 | Coronavac | 3 | 84 (NP) | 180 (NP) | 608.5 (513.75, 734.75) | 0.74 (0.62, 1.12) | All | NP |
| Wang *et al.* | 2024 | China | Cohort study | 228 | 129 | 34 (29, 40) | 40 (31.5, 51) | 9/219 | 25/104 | Coronavac | 3 | 180 (NP) | 240 (NP) | NP | NP | 91.23% | < 50 |
| Tan *et al.* | 2024 | China | A cross-sectional study | 687 | 1222 | 34 (29, 42) | 35 (30, 44) | 107/580 | 738/484 | NP | 3 or 4 | 126 (NP) | 218 (NP) | 424.5 | NP | 97.10% | < 50 |
| Springer *et al.* | 2024 | USA | Cohort study | 36 | 15 | 55 (44, 77) | NP | 6/30 | NP | BNT162b mRNA-1273 | 3 or 4 | 54 (NP) | 218 (NP) | NP | NP | All | NP |
| Ngare *et al.* | 2024 | Japan | Cohort study | 18 | NP | 49 (36, 66) | NP | 2/80 | NP | BNT162b  mRNA-1273 | 3 | 51 (NP) | 207 (NP) | 470 (314, 643) | 0.9 (0.4, 1.1) | All | < 50 |
| Montesi *et al.* | 2024 | Italy | Cohort study | 497 | NP | 54 (44, 59) | NP | 89/408 | NP | Spikevax™ | 3 or 4 | 176 (NP) | NP | 701 (512, 934) | 0.81 (0.56, 1.14) | All | NP |
| Matusali *et al.* | 2024 | Italy | Cohort study | 11 | 14 | 58 (53, 64) | 47 (39, 58) | NP | NP | Raxtozinameran | 3 | 31 (NP) | 384 (NP) | 763  (432, 1031) | NP | NP | NP |
| Matsumoto *et al.* | 2024 | Japan | Cohort study | 34 | NP | 45 (40, 51) | NP | 0/34 | NP | BNT162b2 mRNA-1273 NVX-cov2373 | NP | 171 (NP) | NP | 521 (405, 708) | 0.74 (0.41, 1.17) | All | NP |
| Liu *et al.* | 2024 | China | Cohort study | 1427 | NP | 40 (34, 48) | NP | 1395/32 | NP | mRNA-1273 BNT162b2 | 3 | NP | 90 (NP) | 631 (476, 813) | NP | 96.50% | NP |
| Datwani *et al.* | 2024 | Canada | Cohort study | 50 | 87 | 58 (42, 65) | 50 (35,72) | 6/44 | 58/29 | mRNA-1273 BNT162b2 | 3 | NP | 182 (NP) | 695 (468, 983) | NP | NP | < 50 |
| Chittrakarn *et al.* | 2024 | Thailand | Cohort study | 30 | NP | 47 (43.5, 50.5) | NP | 12/18 | NP | Coronavac Vaxzevria | 3 | 84 (NP) | NP | 575 (476.5, 681) | NP | All | 139 (110, 156) |
| Cherneha *et al.* | 2024 | German | Cohort study | 29 | 14 | 56.7 (53.3, 61.1) | 32 (26, 58) | 2/27 | 8/6 | BNT162b2 | 3 | NP | NP | 746 (583, 943) | NP | All | < 20 |
| Zhang *et al.* | 2023 | China | Cohort study | 165 | NP | >20 | NP | 6/159 | NP | BBIBP-corv | 3 | 45 (NP) | 120 (NP) | NP | NP | All | < 50 |
| Zhan *et al.* | 2023 | China | A cross-sectional study | 318 | 241 | 35 (30, 41) | NP | 24/294 | NP | Coronavac | 3 | 135 (NP) | 180 (NP) | 553 (410, 667) | NP | All | < 50 |
| Zeng *et al.* | 2023 | China | Cohort study | 65 | NP | 34 (30, 39) | NP | 62/3 | NP | BBIBP-corv coronavac | 3 | 35 (NP) | 223 (NP) | 522 (417.5, 674.5) | NP | All | < 50 |
| Yu *et al.* | 2023 | China | Cohort study | 78 | 30 | 41 (34.5, 47) | NP | 9/68 | 21/9 | Coronavac | 3 | NP | NP | 387 (232, 549) | 0.73 (0.62, 1.01) | All | < 50 |
| Yi *et al.* | 2023 | China | Cohort study | 41 | NP | 30 (19, 60) | NP | 1/40 | NP | Coronavac Covilo | 3 | 100 (NP) | NP | 403 (313, 827) | 0.50 (0.30, 0.79) | All | NP |
| Yang *et al.* | 2023 | China | Cohort study | 47 | 30 | 34.7 (31.0, 38.2) | 38.5 (35.0, 48.5) | 4/43 | 5/25 | Coronavac | 3 | 84 (NP) | NP | 597.5 (483.2, 814.7) | 0.74 (0.61, 1.15) | All | NP |
| Vicenti *et al.* | 2023 | Italy | Cohort study | 100 | NP | 54 (49, 60) | NP | 17/83 | NP | BNT162b2 | 3 | NP | 180 (NP) | 580 (411, 786) | NP | All | 58 (49, 72) |
| Vergor *et al.* | 2023 | Italy | Cohort study | 314 | NP | NP | NP | 55/259 | NP | BNT162b2 mRNA-1273 | 3 or 4 | 14 (NP) | 120 (NP) | NP | NP | All | < 50 |
| Tau *et al.* | 2023 | Israel | Cohort study | 45 | NP | 48 (40, 53) | NP | 8/37 | NP | BNT162b2 | 3 | 150 (NP) | 180 (NP) | 660 (515, 958) | 0.27 | All | NP |
| Swan *et al.* | 2023 | Canada | Cohort study | 73 | 17 | 42 (30, 53) | 29 (23, 37) | 42/31 | 12/5 | Pfizer-biontech Comirnaty or Moderna Spikevax | 3 | 53 (NP) | 189 (NP) | NP | NP | All | NP |
| Ray *et al.* | 2023 | Sweden | Cohort study | 68 | 75 | 54 (33, 65.75) | 52 (43.5, 63) | 28/40 | 43/32 | BNT162b2 mRNA-1273 | 3 | NP | NP | 615 (290, 730) | 0.95 (0.47, 1.35) | All | < 50 |
| Qu *et al.* | 2023 | China | Cohort study | 38 | NP | 40 (36, 56) | NP | 2/36 | NP | BBIBP-corv ZF2001 | 3 | 180 (NP) | NP | 426 (305, 580) | 0.73 (0.58, 0.89) | All | < 20 |
| Matveev *et al.* | 2023 | Canada | Cohort study | 68 | 23 | 63 (58.0, 69.0) | 62 (58.0, 70.0) | 1/67 | 23 | BNT162b2 mRNA-1273 chadox1 | 3 | 130 (NP) | NP | 527 (364, 665) | 0.9 (0.5, 1.2) | All | < 40 |
| Marchitto *et al.* | 2023 | Canada | Cohort study | 40 | NP | 51 (25, 77) | NP | 7/33 | NP | BNT162b2 mRNA-1273 | 3 | 84 (NP) | 112 (NP) | NP | NP | All | NP |
| Malin *et al.* | 2023 | Germany | Cohort study | 76 | NP | 55 (44, 59) | NP | 21/66 | NR | BNT162b2 mRNA-1273 | 3 | 22 (NP) | NP | 670 (540, 850) | NP | All | < 50 |
| Lv *et al.* | 2023 | China | Cohort study | 34 | 34 | 43.5 (37.75, 47.25) | 30 (22.75, 39.00) | 16/18 | 17/17 | Coronavac BBIBP-corv | 3 | 17 (NP) | NP | NP | NP | All | < 50 |
| Lu *et al.* | 2023 | China | A cross-sectional study | 100 | NP | 57 (46, 64) | NP | 37/63 | NP | Coronavac BBIBP-corv | 3 | 98 (NP) | 152 (NP) | 362 (227, 501) | NP | All | < 50 |
| Loubet *et al.* | 2023 | China | Cohort study | 522 | NP | 52.5 (41.9, 61.3) | NP | 0/522 | NP | BNT162b2 mRNA-1273 | 3 | 30 (NP) | NP | NP | NP | All | NP |
| López-Cortés *et al.* | 2023 | Spain | Cohort study | 46 | NP | 48 (42, 53) | NP | 10/36 | NP | Mrna-1273/BNT162b2 | 3 | 30 (NP) | 204 (NP) | 654 (512, 849) | 1.12 (0.85, 1.50) | All | NP |
| Li *et al.* | 2023 | China | Cohort study | 57 | 50 | 35 (30, 40) | NP | NP | NP | Coronavac Covilo | 3 | 14 (NP) | 204 (NP) | 668.8 (442.0, 895.6) | 1.18 (0.6, 1.76) | All | NP |
| Lapointe *et al.* | 2023 | Canada | Cohort study | 99 | 152 | 54 (40, 61) | 47 (35, 70) | 11/88 | 102/50 | mRNA-1273/BNT162b2 | 3 | 30 (NP) | 80 (NP) | 715 (545, 943) | NP | All | < 50 |
| Kling *et al.* | 2022 | America | Cohort study | 140 | 75 | 56.5 (55, 58) | NP | 7/133 | 43/32 | mRNA-1273/BNT162b2 Ad26.COV2.S | 3 | NP | NP | 760 | NP | NR | < 50 |
| Jin *et al.* | 2023 | China | Cohort study | 45 | 31 | 35 (31.0, 39.5) | 39 (35.0, 48.0) | 2/43 | 8/23 | NP | 3 | 84 (NP) | 180 (NP) | 587 (505, 807) | 0.76 (0.57, 1.04) | All | NP |
| Hung *et al.* | 2023 | China | Cohort study | 72 | 362 | >20 | >20 | 1/71 | 271/91 | mRNA-1273 and chadox1 | 3 | NP | 110 (NP) | NP | NP | All | NP |
| Heftdal *et al.* | 2023 | Denmark | Cohort study | 378 | 224 | 56 (48, 63) | 55 (44, 63) | 42/336 | 14/210 | BNT162b2 | 3 | 328 (NP) | NP | 640 (490, 800) | NP | 99.70% | < 50 |
| Hassold *et al.* | 2023 | France | Cohort study | 23 | NP | 56 (52.9, 66.8) | NP | 7/16 | NP | BNT162b2/ mRNA-1273/chadox1-ncov19 | 3 | NP | NP | 556 (286, 726) | NP | All | NP |
| Fusco *et al.* | 2023 | Italy | Cohort study | 156 | NP | 51 | NP | 21/214 | NP | BNT162b2 | 3 | NP | NP | 686 | 1 (NP) | All | < 50 |
| Fidler *et al.* | 2023 | United Kingdom | Cohort study | 43 | NP | 42.5 (37.2, 49.8) | NP | 0/43 | NP | Chadox1 | 3 | 182 (NP) | NP | 694 (573.5, 859.5) | NP | All | NP |
| El Moussaoui *et al.* | 2023 | Belgium | Cohort study | 80 | 51 | 45.6 (34.9, 56.3) | 43 (33, 53) | 37/43 | 40/11 | BNT162b2 mRNA-1273 | 3 | 25 (NP) | 49 (NP) | 743 (592, 940) | 1.1 (NP) | All | < 50 |
| Corma-Gómez *et al.* | 2023 | Spain | Case-control studies | 54 | NP | 57 (52, 64) | NP | 6/48 | NP | NP | 3 | NP | NP | 283 (147, 541) | NP | All | < 50 |
| Cheung *et al.* | 2023 | Canada | Cohort study | 63 | NP | 57 (44, 65) | NP | 9/54 | NP | BNT162b2 mRNA-1273 | 3 or 4 | NP | 182 (NP) | 720 (540, 920) | NP | All | NP |
| Basso *et al.* | 2022 | Italy | Cohort study | 184 | NP | 38 (30, 45) | NP | 32/152 | NP | BNT162b2 | 3 | 180 (NP) | 639 (NP) | NP | NP | All | < 50 |
| Alexandrova *et al.* | 2023 | Canada | Cohort study | 38 | 24 | 43 (36, 57) | 44 (38, 56) | 5/33 | 12/12 | NP | 3 | 30 (NP) | NP | 700 (480, 839) | 0.81 (0.59, 1.01) | All | NP |
| Vergori *et al.* | 2022 | Italy | Cohort study | 216 | 98 | 54 (47, 59) | NP | 39/177 | NP | BNT162b2 mRNA-1273 | 3 | NP | 81 (NP) | 45 (20, 122) | NP | All | < 50 |
| Tortellini *et al.* | 2022 | Italy | Cohort study | 37 | 18 | 61 (48, 68) | 30 (30, 53) | 11/26 | 5/13 | BNT162b2 | 3 | NP | NP | 547 (308, 714) | NP | All | 40 (40, 166) |
| Tan *et al.* | 2022 | China | Cohort study | 41 | 18 | 38 (33, 47) | 32 (30, 45) | 4/37 | 3/15 | Verocell | 3 | NP | 28 (NP) | 542 (422, 643) | NP | NP | NP |
| Park *et al.* | 2022 | Republic of Korea | Cohort study | 29 | 216 | 44 (34, 56) | 35 (26, 45) | 1/28 | 76/38 | BNT162b2; mRNA1273 | 3 | NP | NP | 670 (527.1, 830.3) | NP | NP | < 50 |
| Moussaoui *et al.* | 2022 | Belgium | Cohort study | 80 | 51 | 45.2 (34.6, 55.8 | 43.7 (32.2, 55.2) 11.5 | 37/43 | NP | BNT162b2; mRNA-1273 | 3 | 17 (NP) | 189 (NP) | 743 (592, 940) | 1.1 (0.53, 1.67) | All | < 50 |
| Lamacchia *et al.* | 2022 | Italy | Cohort study | 8 | NP | 54 | NP | NP | NP | BNT162b2 | 3 or 4 | NP | NP | 1300 | 1.7 (NP) | All | NP |
| Gianserra *et al.* | 2022 | Italy | Cohort study | 42 | NP | 53 (48, 61) | NP | 16/37 | NP | BNT162b2 | 3 | NP | NP | 687 (488, 929) | NP | 42.90% | < 50 |
| Bessen *et al.* | 2022 | Germany | Cohort study | 71 | 20 | 46.1 (35.2, 57) | 39.4 (27.5, 51.3） | 9/62 | 12/8 | BNT162b2; mRNA-1273 | 3 | NP | 166 (NP) | NP | NP | NP | < 50 |

PWH: people with HIV. HC: healthy controls. F/M: female/male. ART: antiretroviral therapy. NP: not provided. IQR: interquartile range.

**Supplementary Table S3. Risk of bias of all included cohort studies using the Newcastle-Ottawa quality assessment scale.**

| **Author** | **Year** | **Selection** | | | | **Comparability** | **Outcome** | | | **Total score** | **Risk level^#^** |
| --- | --- | --- | --- | --- | --- | --- | --- | --- | --- | --- | --- |
|  |  | **Representativeness of the Exposed Cohort** | **Selection of the Non-Exposed Cohort** | **Ascertainment of Exposure** | **Demonstration That Outcome of Interest Was Not Present at Start of Study** |  | **Assessment of Outcome** | **Was Follow-Up Long Enough for Outcomes to Occur** | **Adequacy of Follow Up of Cohorts** |  |  |
| Wu *et al.* | 2024 | 1 | 1 | 1 | 0 | 0 | 1 | 0 | 1 | 6 | Moderate |
| Wang (1) *et al.* | 2024 | 1 | 0 | 1 | 1 | 1 | 0 | 1 | 1 | 6 | Moderate |
| Wang *et al.* | 2024 | 1 | 1 | 0 | 1 | 1 | 0 | 1 | 1 | 6 | Moderate |
| Springer *et al.* | 2024 | 1 | 1 | 0 | 1 | 0 | 1 | 1 | 0 | 5 | Moderate |
| Ngare,*et al.* | 2024 | 1 | 0 | 0 | 1 | 0 | 0 | 1 | 0 | 2 | High |
| Montesi *et al.* | 2024 | 1 | 0 | 1 | 0 | 1 | 0 | 1 | 1 | 5 | Moderate |
| Matusali *et al.* | 2024 | 1 | 1 | 0 | 1 | 0 | 1 | 0 | 1 | 5 | Moderate |
| Matsumoto *et al.* | 2024 | 1 | 1 | 0 | 1 | 1 | 0 | 1 | 1 | 6 | Moderate |
| Liu *et al.* | 2024 | 1 | 0 | 1 | 1 | 1 | 0 | 0 | 1 | 4 | High |
| Datwani *et al.* | 2024 | 1 | 1 | 1 | 1 | 1 | 0 | 0 | 1 | 6 | Moderate |
| Chittrakarn *et al.* | 2024 | 1 | 1 | 0 | 1 | 1 | 0 | 0 | 1 | 5 | Moderate |
| Cherneha *et al.* | 2024 | 1 | 0 | 1 | 1 | 1 | 1 | 1 | 0 | 6 | Moderate |
| Zhang *et al.* | 2023 | 1 | 0 | 1 | 1 | 1 | 1 | 1 | 0 | 6 | Moderate |
| Zeng *et al.* | 2023 | 1 | 0 | 1 | 0 | 0 | 1 | 1 | 0 | 4 | High |
| Yu *et al.* | 2023 | 1 | 1 | 1 | 1 | 0 | 0 | 1 | 1 | 6 | Moderate |
| Yi *et al.* | 2023 | 1 | 0 | 1 | 1 | 0 | 1 | 1 | 0 | 5 | Moderate |
| Yang *et al.* | 2023 | 1 | 1 | 1 | 0 | 0 | 1 | 1 | 1 | 6 | Moderate |
| Vicenti *et al.* | 2023 | 1 | 1 | 1 | 1 | 0 | 1 | 1 | 0 | 6 | Moderate |
| Vergor *et al.* | 2023 | 1 | 0 | 1 | 1 | 1 | 0 | 1 | 0 | 5 | Moderate |
| Tau *et al.* | 2023 | 1 | 0 | 1 | 1 | 1 | 0 | 0 | 1 | 5 | Moderate |
| Swan *et al.* | 2023 | 1 | 1 | 1 | 1 | 1 | 0 | 0 | 1 | 6 | Moderate |
| Ray *et al.* | 2023 | 1 | 0 | 1 | 1 | 1 | 1 | 1 | 0 | 6 | Moderate |
| Qu *et al.* | 2023 | 1 | 0 | 1 | 1 | 1 | 1 | 1 | 0 | 6 | Moderate |
| Matveev *et al.* | 2023 | 1 | 1 | 1 | 1 | 0 | 1 | 1 | 0 | 6 | Moderate |
| Marchitto *et al.* | 2023 | 1 | 0 | 1 | 1 | 0 | 1 | 1 | 0 | 5 | Moderate |
| Malin *et al.* | 2023 | 1 | 0 | 1 | 1 | 0 | 1 | 1 | 0 | 5 | Moderate |
| Lv *et al.* | 2023 | 1 | 1 | 0 | 1 | 1 | 0 | 1 | 0 | 5 | Moderate |
| Loubet *et al.* | 2023 | 1 | 0 | 1 | 1 | 1 | 0 | 0 | 0 | 4 | High |
| López-Cortés *et al.* | 2023 | 1 | 0 | 1 | 1 | 1 | 0 | 0 | 1 | 5 | Moderate |
| Li *et al.* | 2023 | 1 | 1 | 1 | 1 | 1 | 1 | 1 | 1 | 8 | Low |
| Lapointe *et al.* | 2023 | 1 | 1 | 1 | 1 | 0 | 1 | 1 | 0 | 6 | Moderate |
| Kling *et al.* | 2023 | 1 | 1 | 1 | 1 | 0 | 0 | 1 | 0 | 5 | Moderate |
| Jin *et al.* | 2023 | 1 | 1 | 0 | 1 | 1 | 0 | 0 | 1 | 5 | Moderate |
| Hung *et al.* | 2023 | 1 | 1 | 0 | 1 | 0 | 0 | 1 | 0 | 4 | Moderate |
| Heftdal *et al.* | 2023 | 1 | 1 | 1 | 1 | 0 | 0 | 1 | 1 | 6 | Moderate |
| Hassold *et al.* | 2023 | 1 | 0 | 1 | 0 | 1 | 1 | 1 | 0 | 5 | Moderate |
| Francesco *et al.* | 2023 | 1 | 0 | 0 | 1 | 1 | 1 | 1 | 0 | 5 | Moderate |
| Fidler *et al.* | 2023 | 1 | 0 | 1 | 1 | 1 | 1 | 0 | 0 | 5 | Moderate |
| Moussaoui *et al.* | 2023 | 1 | 1 | 1 | 1 | 1 | 0 | 0 | 1 | 6 | Moderate |
| Costiniuk *et al.* | 2023 | 1 | 1 | 1 | 1 | 1 | 0 | 1 | 1 | 7 | Low |
| Cheung *et al.* | 2023 | 1 | 1 | 0 | 1 | 1 | 0 | 0 | 1 | 5 | Moderate |
| Basso *et al.* | 2023 | 1 | 0 | 1 | 1 | 1 | 0 | 1 | 1 | 6 | Moderate |
| Alexandrova *et al.* | 2023 | 1 | 1 | 0 | 1 | 1 | 0 | 1 | 1 | 6 | Moderate |
| Vergori *et al.* | 2022 | 1 | 0 | 1 | 1 | 1 | 0 | 1 | 1 | 6 | Moderate |
| Tortellini *et al.* | 2022 | 1 | 0 | 1 | 1 | 1 | 0 | 1 | 1 | 6 | Moderate |
| Park *et al.* | 2022 | 1 | 1 | 1 | 1 | 1 | 0 | 1 | 0 | 6 | Moderate |
| Moussaoui *et al.* | 2022 | 1 | 1 | 1 | 1 | 1 | 1 | 1 | 0 | 7 | Low |
| Lamacchia *et al.* | 2022 | 1 | 1 | 0 | 1 | 1 | 0 | 1 | 0 | 5 | Moderate |
| Gianserra *et al.* | 2022 | 1 | 0 | 1 | 1 | 1 | 0 | 1 | 0 | 5 | Moderate |
| Bessen *et al.* | 2022 | 1 | 1 | 0 | 1 | 1 | 0 | 1 | 0 | 5 | Moderate |

^#^Low (total score ≥ 7), moderate (total score 5-6), and high (total score ≤ 4) risk of bias.

**Supplementary Table S4. Risk of bias of all included case-control studies using the Newcastle-Ottawa quality assessment scale.**

| **Author** | **Year** | **Selection** | | | | **Comparability** | **Outcome** | | | **Total score** | **Risk level^#^** |
| --- | --- | --- | --- | --- | --- | --- | --- | --- | --- | --- | --- |
|  |  | **Representativeness of the exposed cohort** | **Selection of the non-exposed cohort** | **Ascertainment of exposure** | **Demonstration that outcome of interest was not present at start of study** |  | **Assessment of outcome** | **Was follow-up long enough for outcomes to occur** | **Adequacy of follow up of cohorts** |  |  |
| Corma-Gómez *et al.* | 2023 | 1 | 1 | 0 | 1 | 1 | 0 | 1 | 0 | 5 | Moderate |

^#^Low (total score ≥ 7), moderate (total score 5-6), and high (total score ≤ 4) risk of bias.

**Supplementary Table S5. Risk of bias of all included a cross-sectional study using the Agency for Healthcare Research and Quality.**

| **Author** | **Year** | **Define the source of information (survey, record review)** | **List inclusion and exclusion criteria for exposed and unexposed subjects (cases and controls) or refer to previous publications** | **Indicate time period used for identifying patients** | **Indicate whether or not subjects were consecutive if not population-based** | **Indicate if evaluators of subjective components of study were masked to other aspects of the status of the participants** | **Describe any assessments undertaken for quality assurance purposes (e.g., test/retest of primary outcome measurements)** | **Explain any patient exclusions from analysis** | **Describe how confounding was assessed and/or controlled** | **If applicable, explain how missing data were handled in the analysis** | **Summarize patient response rates and completeness of data collection** | **Clarify what follow-up, if any, was expected and the percentage of patients for which incomplete data or follow-up was obtained** | **Total score** | **Quality level^#^** |
| --- | --- | --- | --- | --- | --- | --- | --- | --- | --- | --- | --- | --- | --- | --- |
| Tan *et al.* | 2024 | 1 | 1 | 0 | 0 | 1 | 0 | 0 | 0 | 0 | 0 | 0 | 3 | Low |
| Zhan *et al.* | 2023 | 1 | 0 | 0 | 0 | 1 | 0 | 0 | 0 | 1 | 0 | 0 | 3 | Low |
| Lu *et al.* | 2023 | 1 | 0 | 0 | 1 | 1 | 1 | 0 | 0 | 1 | 1 | 0 | 6 | Moderate |

^#^Low quality (total score 0-3), moderate quality (total score 4-7), and high quality (total score 8-11).

**Supplementary Figure Legends**

**Supplementary Figure S1. Sensitivity analysis.**

Note. Immune response rate of PLWH.

**Supplementary Figure S2. Sensitivity analysis.**

Note. Immune response rate among PLWH compared to HC. The results shown are the culling of studies from Matveev.

**Supplementary Figure S3. Publication bias.**

Note. The publication bias of studies on immune response rate among PLWH vs HC after booster dose of COVID-19 vaccine.
