# Supplementary figures and images for: Immunogenicity and safety of the booster COVID-19 vaccine among people with HIV: a systematic review and meta-analysis
Source: Front Immunol. 2025 Sep 17;16:1668576. doi: 10.3389/fimmu.2025.1668576 (PMC12484232; doi:10.3389/fimmu.2025.1668576)

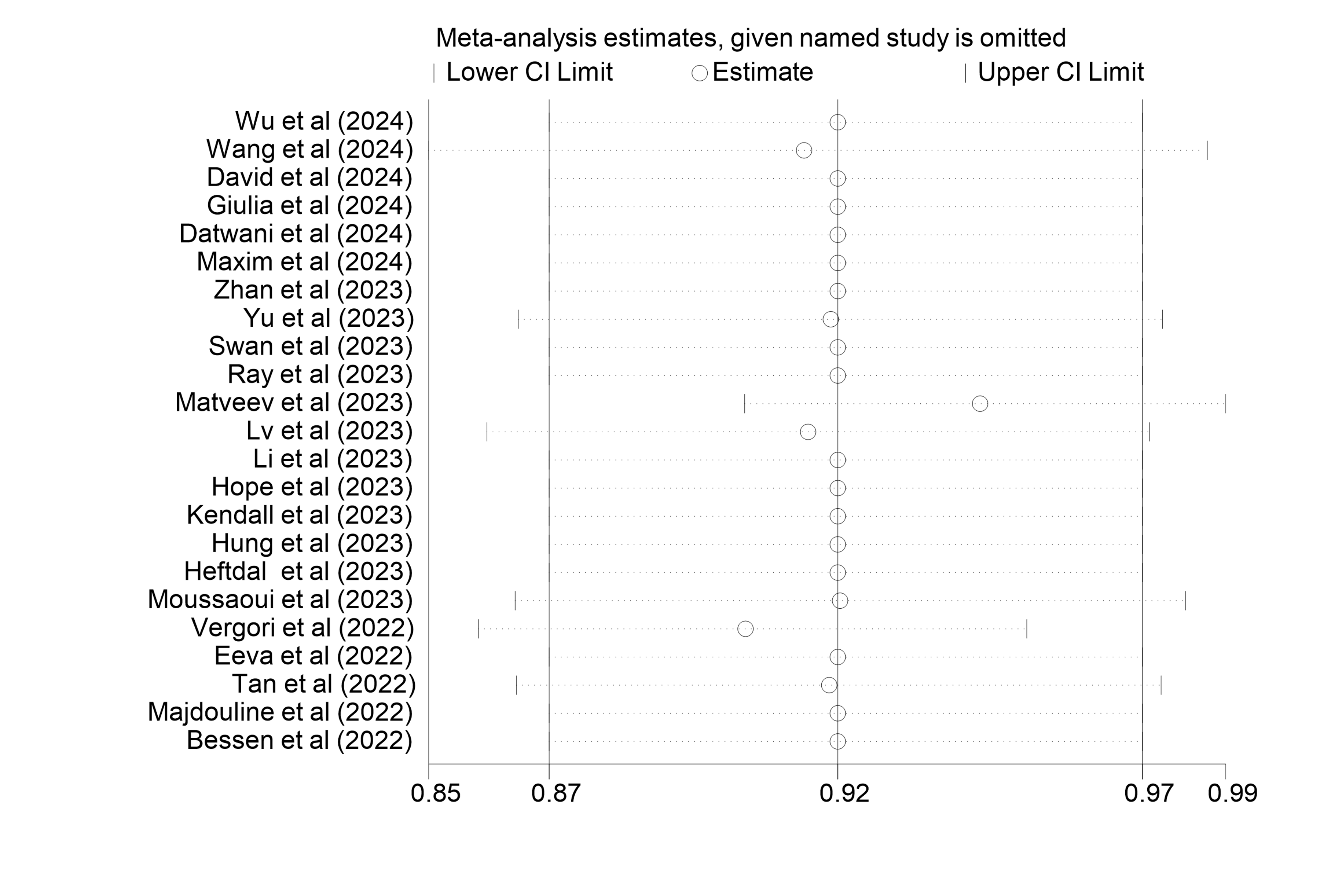

Supplement: Supplementary file 2 [file Image1.tif]

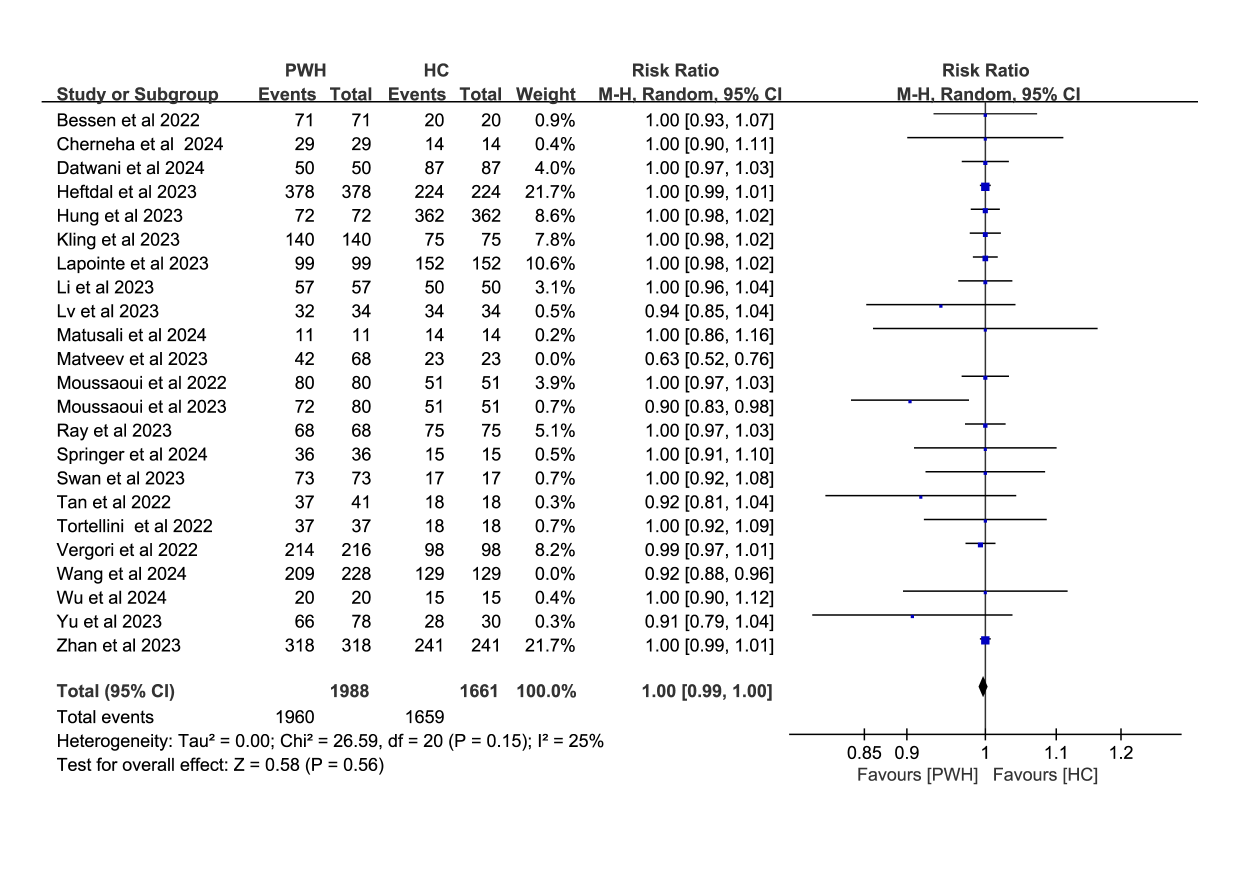

Supplement: Supplementary file 3 [file Image2.tiff]

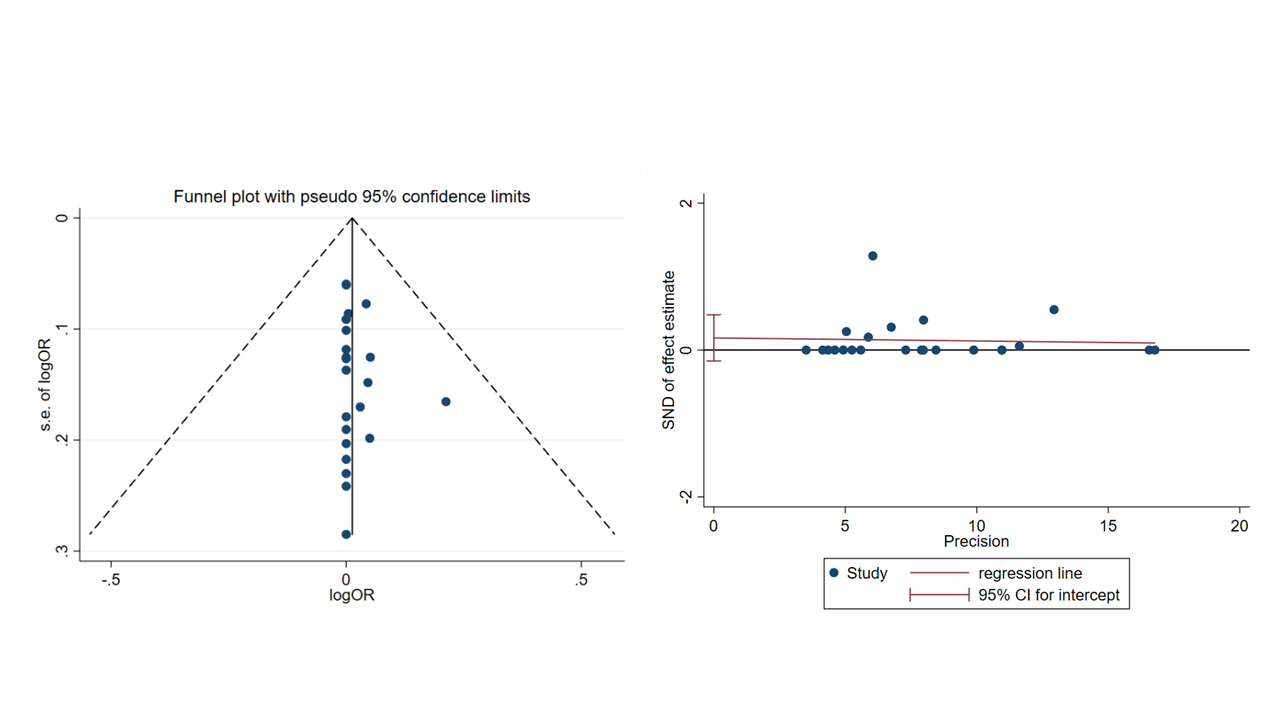

Supplement: Supplementary file 4 [file Image3.tif]
